# Chemical Composition, Lipid-Soluble Bioactive Compounds and Potential Health Benefits of the Moss *Hypnum cupressiforme* Hedw

**DOI:** 10.3390/plants12244190

**Published:** 2023-12-18

**Authors:** Zhana Petkova, Olga Teneva, Ginka Antova, Maria Angelova-Romova, Gana Gecheva, Ivanka Dimitrova-Dyulgerova

**Affiliations:** 1 Department of Chemical Technology, Faculty of Chemistry, University of Plovdiv “Paisii Hilendarski”, 24 Tzar Asen Street, 4000 Plovdiv, Bulgaria; olga@uni-plovdiv.bg (O.T.); ginant@uni-plovdiv.bg (G.A.); maioan@uni-plovdiv.bg (M.A.-R.); 2 Department of Ecology and Environmental Conservation, Faculty of Biology, University of Plovdiv “Paisii Hilendarski”, 24 Tzar Asen Street, 4000 Plovdiv, Bulgaria; ggecheva@uni-plovdiv.bg; 3 Department of Botany and Biological Education, Faculty of Biology, University of Plovdiv “Paisii Hilendarski”, 24 Tzar Asen Street, 4000 Plovdiv, Bulgaria; ivadim@uni-plovdiv.bg

**Keywords:** *Hypnum cupressiforme*, chemical composition, glyceride oil, tocopherols, sterols, fatty acids, lipid indices, health benefits

## Abstract

*Hypnum cupressiforme* Hedw. is the main species for Moss surveys (ICP Vegetation programme) in Southeastern Europe and is widely distributed in the region. In addition to their biomonitoring role, mosses are applied in some countries as a traditional medicine for the treatment of eczema, cuts, burns, eye diseases, etc. Therefore, the chemical and lipid composition of the moss *H. cupressiforme* is of interest to establish their possible application in different fields. The chemical composition of the moss was examined regarding total lipids, proteins, carbohydrates (i.e., fibres), ash, and moisture content. The main lipid-soluble bioactive components were determined as sterols, tocopherols, phospholipids and fatty acids. The major fatty acids were linoleic (14.9%), oleic (13.8%), palmitic (12.5%) and α-linolenic (11.3%) acids. Unsaturated fatty acids (56.4%) prevailed in the glyceride oil, in which the polyunsaturated ones constituted 32.5%. The lipid indices (atherogenicity, thrombogenicity, hypocholesterolemic/hypercholesterolemic ratio, peroxidability, and oxidation stability index) were also theoretically calculated based on the fatty acid composition of the moss lipids to establish their health benefits and the rate of oxidation. The primary results of this study revealed *H. cupressiforme* to be a promising alternative source of bioactive compounds that could be implemented in supplements with health-promoting effects.

## 1. Introduction

Mosses belong to the division of bryophyte species and include over 10,000 species [1,2]. They are highly distributed all around the world, especially in different areas in the Northern Hemisphere [2,3]. Mosses can resist severe climatic conditions as well as endure oxidative stress [1,2]. They do not have true roots, which is why the absorption of water and micro- and macronutrients is carried out by the whole surface [4]. Despite having the ability to serve as bioindicators of air pollution, some of them are also reported to possess an interesting variety of compounds (carbohydrates, lipids, proteins) and a myriad of water- and lipid-soluble bioactive compounds such as polyphenols, organic acids, long-chain fatty acids, sterols, terpenoids, etc. [1,3,5].

*Hypnum cupressiforme* Hedw. is the main species for Moss surveys (ICP Vegetation programme) in Southeastern Europe and is widely distributed in the region. Numerous intraspecific taxa of this species that are distinguished by their leaf shape, size, capsules, colour, etc., are described in the literature. *H. cupressiforme* is a low to medium-sized plant and its colour can vary from light to dark green [6]. Mosses, including *H. cupressiforme*, constitute a part of green infrastructures as a multifunctional material [7]. However, they have also been proven to exhibit antibacterial, antifungal, antitumor, and insecticidal properties due to their unique composition [1]. Anti-neuroinflammatory and neuroprotective properties of *H. cupressiforme* have been studied recently as well [8].

Understanding the chemical and lipid composition of *H. cupressiforme* Hedw. is essential for unravelling its ecological functions, evolutionary adaptations, and its potential applications in both the medicinal and food industries. Yet, to the best of our knowledge, the information on the quantitative amounts of some important macro- and micronutrients as well as lipid-soluble biologically active compounds in *H. cupressiforme* Hedw. is notoriously limited. Scarce information about the fatty acid composition of the glyceride oil from this moss can be found in the literature. For example, Nichols [9] determined that the main fatty acid in *H. cupressiforme* oil was linolenic (23.5%), followed by linoleic (19.7%) and saturated palmitic acid (13.5%). On the other hand, Abay et al. [10] reported a completely different fatty acid composition of the lipids in the same moss, the composition of which was strongly influenced by the type of solvent used for the isolation of the glyceride oil. They revealed that in the oil extracted with hexane, the predominating fatty acids were arachidic (26.52%), palmitic (20.87%), and oleic (19.64%). In the oil obtained by dichloromethane and chloroform were behenic (37.43%) and tetracosanois acids (33.69%). In the oil extracted with ethyl acetate were palmitic (35.41%) and behenic (18.32%). In the extracted with methanol were palmitic (19.96%), linoleic (19.75%), and α-linolenic (15.73%). According to other authors [11], the main fatty acids of *H. cupressiforme* oil were α-linolenic (24.17%), linoleic (22.25%), palmitic (15.28%) and there were significantly high amounts of eicosatrienoic acid (11.32%). Even though, sterol and tocopherol compositions have been analysed in the oils of several other mosses (*Aulacomnium palustre*, *Climacium dendroides*, *Dicranum polysetum*, *Hylocomium splendens*, *Dicranum scoparium*, *Atrichum undulatum*, etc.) [3,12], to the present moment, such information is absent for the oil from *H. cupressiforme*.

For that reason, the current study aimed to examine the chemical composition of the mentioned moss and reveal the most prominent lipid-soluble bioactive components present in its glyceride oil, to establish the health-promoting benefits of *H. cupressiforme*, and expand their application in food and pharmaceutical products.

## 2. Results

### 2.1. Chemical Composition

The chemical composition of plants gives a better understating of their physiology, nutritional value, and ability to resist different atmospheric conditions and oxidative stress. Some components are strongly related to specific functions. For instance, lipids are involved in the regulation of cell metabolism, carbohydrates are important sources of energy, and proteins can partake in biosynthesis, photosynthesis, transportation, etc. [13,14,15]. The number of other components of the chemical composition (such as ash and moisture contents) are mostly influenced by the environmental conditions, soils, pollution, etc. That is why establishing the main components of the chemical composition of the moss *Hypnum cupressiforme* Hedw. is of great importance. The analyses performed revealed the amounts of the major constituents in the investigated moss tissues were glyceride oil, total proteins, and carbohydrates, i.e., fibres, ash, and moisture content (Table 1).

The moss *H. cupressiforme* was characterized by a very high content of carbohydrates (76.41%), a notably low concentration of proteins (7.18%), and a significantly small amount of glyceride oil (which is slightly above 1%). The content of fibres had a remarkably big share of the total carbohydrates, about 40% of all carbohydrates, while their quantity in the whole tissue was 30.81%. The content of total ash and moisture were 3.36% and 11.78%, respectively. Based on the chemical composition of the moss, the energy value of the tissues from *H. cupressiforme* was calculated. Because of the very low levels of glyceride oil, the energy value was calculated as 346 kcal/100 g or 1469 kJ/100 g.

### 2.2. Lipid Composition

Lipids are responsible for many functions in plants, the main being regulation of cell metabolism, but they are part of cell membranes as well. Some of the lipid components are bioactive compounds (such as tocopherols, phytosterols, phospholipids, fatty acids, etc.) that play an important role in plant organisms, mosses in particular, and may exert antioxidant activity (e.g., tocopherols). For that reason, it was essential to determine the lipid composition of *H. cupressiforme*.

The content of the lipid-soluble biologically active components present in the moss tissues and in the isolated glyceride oil is given in Table 2.

Sterols and tocopherols are two of the main components of the unsaponifiable matter of the glyceride oils and the whole tocopherol content of the moss lipids is represented solely by α-tocopherol. Other tocopherol isomers (β-, γ- or δ-tocopherol) were not identified in the oil from *H. cupressiforme*. The oil from *H. cupressiforme* was characterized by relatively high levels of total sterols (3.2%) and extremely high content of tocopherols (1848.5 mg/kg) and phospholipids (50.1%).

By calculating their amounts in the moss tissues (based on the oil content), it was observed that the same components were as follows: 0.041% for the sterols, 23.9% for the tocopherols, and 0.7% for the phospholipids. The reason for this was the small amount of glyceride oil in *H. cupressiforme*.

Individual phospholipid composition was also identified in the lipids from *H. cupressiforme* (Table 3).

Phospholipids are one of the polar lipids present in the lipids. Six individual phospholipids were identified in the moss. The main ones were phosphatidylcholine (19.9%), phosphatidylinositol (19.8%), and phosphatidylserine (17.0%). The other components of the phospholipids were also present in rather significant amounts from 15.1% (phosphatidic acids) to 15.7% (phosphatidylethanolamine). There were also notable quantities of lysophosphatidylcholine (12.5%) which was probably due to the commencing hydrolysis process in the moss tissues and especially in their lipids.

Other extremely important compounds in glyceride oils are the fatty acids, principally the long-chain essential fatty acids. The fatty acid composition of the glyceride oil from moss *H. cupressiforme* is shown in Table 4.

Fatty acids can be divided into saturated (SFA) and unsaturated (UFA), and mono- (MUFA) and polyunsaturated fatty acids (PUFAs), depending on the presence or the absence of double bonds in their hydrocarbon chain.

In the glyceride oil from *H. cupressiforme*, 24 fatty acids were identified, 13 of them were SFAs, and 11 were UFAs. Of the latter, six were MUFAs and five were PUFAs.

Of the SFAs, palmitic acid (C_16:0_) was the major one (12.5%), followed by caprylic acid (C_8:0_, 8.6%), which was a medium-chain fatty acid. In relatively high percentages, stearic acid (C_18:0_, 6.0%) and myristic acid (C_14:0_, 4.4%) were identified. The remaining SFAs were present in considerably small quantities from 0.6% (C_10:0_—capric acid) to 2.7% (C_22:0_—behenic acid). The total percentage of SFAs in the lipids from *H. cupressiforme* accounted for 43,6%, which was almost half of all fatty acids in the oil.

The levels of the UFAs were 56.4% and the amount of MUFAs was lower (23.9%) than that of PUFAs (32.5%). The predominating fatty acid of the MUFA was oleic (C_18:1_, 13.8%) while the quantity of the others was from 0.9% (C_14:1_—myristoleic acid) to 3.2% (C_15:1_—pentadecenoic acid). The fatty acids in the highest levels from the PUFA were linoleic (C_18:2_ (n-6), 14.9%) and α-linolenic acid (C_18:3_ (n-3), 11.3%). The other fatty acids identified in significantly high amounts were arachidonic acid (C_20:4_ (n-6), 3.3%) and γ-linolenic acid (C_18:3_ (n-6), 2.4%), while the fatty acid in the lowest percentage of PUFAs was dihomo-γ-linolenic acid (C_20:3_ (n-6)), the amount of which accounted for 0.6%.

**Figure 1:**
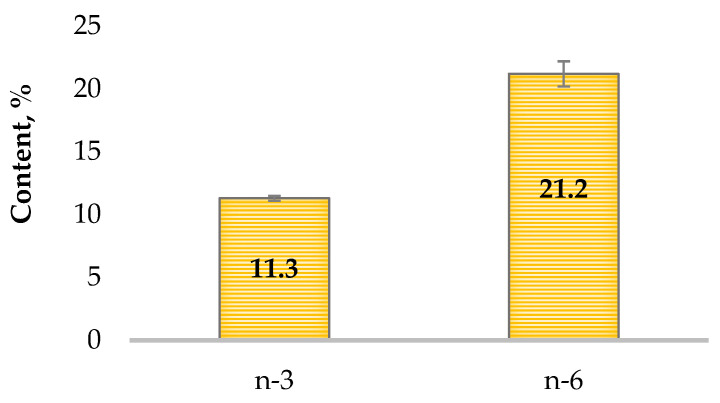
Content of n-3 and n-6 fatty acids in the lipids isolated from the moss *Hypnum cupressiforme*.

The percentages of the total n-3 and n-6 fatty acids present in the oil from *H. cupressiforme* are given in Figure 1. The only n-3 fatty acid identified in the glyceride oil was α-linolenic acid, the amount of which was 11.3%, while from the n-6 fatty acids, linoleic, γ-linolenic, dihomo-γ-linolenic, and arachidonic acids were present and their content accounted for 21.2%. The optimal recommended ratio of n-6 to n-3 fatty acids in the diet to exert some health-promoting effects was established to be 1:1–2:1 [16], which was in accordance with the ratio in the glyceride oil from *H. cupressiforme* that was established to be 1.88:1.

### 2.3. Lipid Indices

Several very important lipid indices can be calculated based on the identified individual fatty acid composition. Some of them indicate possible health benefits of the glyceride oils, such as anti-atherogenic, anti-thrombogenic properties, etc. Others may reveal the rate of oxidation of the lipids, their stability while exposed to atmospheric conditions, and their ability to be stable upon oxidative stress.

Table 5 shows the main lipid indices of the glyceride oil from moss *H. cupressiforme* and the key interpretations of the optimal values and their significance for the lipids are given.

The Iodine value is calculated based on the amounts (in %) of the oleic, linoleic, and linolenic acids. Its value for the examined moss was 76.9 gI_2_/100 g which placed that oil in the group of the non-drying glyceride oils which were not able to form a layer when they were exposed to air.

Another significant indicator is the ratio of PUFA to SFA, the optimal values of which are established to be between 0.8 and 1.2 [18]. In the investigated glyceride oil from *H. cupressiforme*, this ratio was slightly lower (0.7454) but still very close to the recommended one.

The indices of atherogenicity and thrombogenicity indicate the healthier properties of the lipids and the lower the values, the greater the anti-atherogenic and anti-thrombogenic effects are exerted by the oils. In this regard, the values of the indices of the *H. cupressiforme* oil were low at 0.5709 for the index of atherogenicity and 0.4019 for the index of thrombogenicity, which meant that it might exert beneficial effects.

On the contrary, the higher the values of the hypocholesterolemic to hypercholesterolemic (HH) ratio, the better the hypocholesterolemic potential of the lipids is observed. The value of this ratio in *H. cupressiforme* oil was established to be 2.4368, which also could be an indicator of its health-promoting impact.

The rest of the indicators are focused on the stability of the oils: peroxidability index (PI), an allylic position equivalent (APE), a bis-allylic position equivalent (BAPE), and an oxidation stability index (OSI). The values of PI help predict the relative oxidation stability of the lipids. The calculated value of this indicator for *H. cupressiforme* oil was 57.3 which was due to the more significant amount of monoenic, dienoic, and trienoic acids in this oil compared to tetraenoic acids, and the lack of pentaenoic and hexaenoic fatty acids. An APE indicates the presence of allylic groups in the hydrocarbon chain of the fatty acids, while BAPE gives information about the presence of hydrogen atoms in a bis-allylic position [22]. These indicators for the glyceride oil from *H. cupressiforme* were 84.8 and 42.3, respectively. The higher the values of APE and BAPE, the higher the susceptibility of the oil to oxidation. The OSI can be used for predicting the shelf-life of the oils and its value for the examined moss oil is relatively low (2.0).

Based on the given results, it is obvious that the moss *H. cupressiforme* is a source of macro- and micronutrients as well as being rich in concentrated amounts of lipid-soluble biologically active compounds with beneficial effects.

## 3. Discussion

In the past decade, numerous studies have focused on various plant species that are not examined in detail, yet, they may contain unique bioactive compounds that can be isolated and find a niche in various industries, including their use in pharmaceutical products. In this respect, mosses are very suitable for exploration because of their wide distribution worldwide and ability to resist great temperature amplitudes. *H. cupressiforme* is the most widespread in Southeastern Europe and, besides its biomonitoring role, it is utilised for medicinal purposes as well. Nevertheless, this moss has not been thoroughly examined, which was the motivation for the current study.

The composition of *H. cupressiforme* is predominated by carbohydrates whilst the other main compounds (proteins and lipids) are found in low amounts. Several authors reported that the major ingredients of bryophytes were carbohydrates which took an important part in their tolerance toward stress; they were involved in the building of the cell walls and facilitated the interaction with the environment [2,3,24,25]. According to Maksimova et al. [26], up to 60% of the dry matter of most bryophytes was hemicellulose and pectin, while the content of the total proteins was from 5 to 10%, which confirmed the result obtained in the present study. The content of nitrogen in the examined moss *H. cupressiforme* accounted for 1.15%, which was the same for *Sphagnum* mosses that contained up to 1% nitrogen [27]. Klavina et al. [2] also established that the content of this element in several bryophytes (*Aulacomnium palustris* (Hedw.) Schwagr., *Polytrichum commune* Hedw., *Polytrichum juniperum* Hedw., *Ptilium crista-castrensis* (Hedw.) De Not., *Pleurozium schreberi* (Willd. ex Brid.) Mitt., *Rhytidiadelphus triquetrus* (Hedw.) Warnst., *Sphagnum girgensohnii* Russow, *Sphagnum magellanicum* Brid., *Sphagnum capillifolium* (Ehrh.) Hedw., *Sphagnum angustifolium* and *Plagiochila asplenioides*) ranged from 0.4 to 2.0%. Nitrogen is a very important element for various physiological processes and the proper development of plants as well as indirectly being involved in the growth of plant cells [28]. The total amount of lipids in the studied moss *H. cupressiforme* was extremely small and differed from the findings by Klavina [24], who established that their content in some bryophytes was from 5 to 10%. The role of fibres in plants is concentrated on their position as a structural element. Their content in the examined moss was almost twice as high as reported in previous studies which found that cellulose content in several bryophytes ranged from 15 to 25% [24]. The benefits of fibres in the human diet are undeniable. They mainly have an impact on the proper function of the gastrointestinal tract, but also influence lipid metabolism and mineral bioavailability, and reduce the risk of a number of diseases such as hypertension, diabetes, obesity, cardiovascular diseases, etc. [29].

Commisso et al. [30] classified the different lipid compounds of the bryophytes into four classes according to their functions in plants: storage lipids (triacylglycerols, sterol esters), membrane lipids (sterols, phospholipids), surface lipids (waxes), and signalling compounds (phosphoinositides). The present study determined the total sterol content, total and individual tocopherols, phospholipids, and the main fatty acids. The content of the sterols in the glyceride oil was significantly high, but its amount in the whole tissue was very low because the share of the lipids in the moss was extremely small. Other authors have also reported low levels of the total sterols. For example, Chiu et al. [31] found that the sterol content of most of the bryophytes ranged from 0.04% to 0.21% of the tissue (dry weight). On the other hand, most of the plant lipids have been established to possess from 0.15 to 0.90% sterols [32], which is much lower than in the moss glyceride oil. Plant sterols (known as phytosterols) have several nutritional and physiological properties. They are able to lower the levels of cholesterol and low-density lipids (LDL) in blood plasma which have an indirect effect on the prevention of cardiovascular disease [32]. Total tocopherols of the examined moss were in significantly high amounts (over 1500 mg/kg) and the only identified representative was α-tocopherol. In previous studies, it has also been demonstrated that in different bryophyte species, α-tocopherol has been identified, which has antioxidant as well as antifungal properties [30]. Phospholipids, alongside proteins and glycolipids, play an important part in the formation of biological membranes [32]. The share of the phospholipids in the glyceride oil from the moss *H. cupressiforme* was exactly 50% of all lipid components. Several authors also reported high quantities of phospholipids in the total lipids from different moss species. For instance, Dembitsky et al. [33] established that the phospholipid content in the lipids from *Mnium marginatum*, *Anomodon viticulosus*, and *Plagiomnium ellipticum* ranged from 6.71 to 20.41%. In *Peurorium schreberi*, *Dicranum polysetum*, and *Polytrichum juniperinum* it varied from 12.07 to 17.05% [34]. While in thirteen moss species from Southwestern Siberia, it ranged from 8.3 (*Sphagnum squarrosum*) to 30.6% (*Philonotis fontana*) [35].

The main phospholipid classes in the examined moss *H. cupressiforme* were phosphatidylcholine (PC) and phosphatidylinositol (PI) but their quantities were not as high as the amounts of the other phospholipids present in the fraction. PC was also the main component in *Polytrichum juniperinum*, *Peurorium schreberi* and *Dicranum polysetum* but its share was significantly higher–from 33.4 to 58.9% [34]. The reported content of phosphatidylethanolamine (PE) in the same moss species was slightly higher (17.7–25.0%) than in the lipids from *H. cupressiforme* (15.7%). On the other hand, the established quantities of PI and phosphatidylserine (PS) in the literature were significantly lower (8.9–16.6% (together with lysophosphatidylethanolamine) and 0.0–4.2%, respectively) [34] than in the examined moss (19.8 and 17.0%). Dembitsky and Rezanka [35] reported that the content of PC in 13 moss species from Southwestern Siberia ranged from 7.5% (*Sphagnum squarrosum*) to 57.5% (*Climacium dendroides*), the PE content ranged from 9.8% (*Climacium dendroides*) to 27.1% (*Philonotis fontana*), and the PI and PS ranged from 3.4 to 12.6%. The established dissimilarities in the phospholipid composition of *H. cupressiforme* from the data from previous studies are probably due to the interspecies differences.

The main fatty acids in the glyceride oils from the examined moss were linoleic, oleic, palmitic, and α-linolenic acids. Small amounts of arachidonic and γ-linolenic acid were also identified (about 3%). Many authors confirmed that long-chain fatty acids are very typical for a myriad of bryophytes. Linoleic and oleic acids were also the main components in *P. schreberi* (15.47% and 11.95%, respectively) and the content of α-linolenic acid was 11.31% [34]. Pejin et al. [36] observed that the major fatty acids in *Atrichum undulatum* were linoleic (26.80%), palmitic (22.17%), α-linolenic (20.50%), and oleic acid (18.49%), while in *Hypnum andoi* it was palmitic acid (63.48%). On the other hand, several authors reported much higher quantities of arachidonic and eicosapentaenoic acids: 28.6 and 9.8% (*Rhytidiadelphus squarrosus*) [37], 20.9–36.7% and 8.6–23.4% (*E. striatum*, *B. rutabulum*, *B. salebrosum*, *S. purum*, *R. squarrosus*, *R. triquetrus*) [38], and 13.52 and 12.77% (*Brachythecium erythrorrhizon*), respectively [39]. Linoleic, α-linolenic and arachidonic acids are not synthesised in humans and, to fulfil the need, external sources rich in these compounds should be included in the diet. Long-chain polyunsaturated fatty acids that have more than two double bonds in the hydrocarbon chain play an important role in the function of the brain and the metabolism of cholesterol and possess health-promoting effects [40,41]. Linoleic acid has been shown to reduce both body fat and the risk of atherosclerosis, α-linolenic acid influences the regulation of blood pressure, and possesses an anti-inflammatory effect, while arachidonic acid plays an important role in the appropriate functioning of the neurological system and immune processes [41]. The ratio of n-6 to n-3 fatty acids of the glyceride oil from *H. cupressiforme* was in the recommended range (1:1–2:1) [16]. Moreover, the imbalanced intake of n-6 over n-3 fatty acids has been confirmed to cause some chronic diseases (diabetes, cancer, cardiovascular diseases, etc.) [42].

After the identification and quantification of the fatty acids present in *H. cupressiforme* oil, several important lipid indices were calculated. These indices are related to the proper estimation of the possible health-promoting effects of the lipids. The ratio of PUFAs to SFAs represents the potential of lipids to decrease incidents of cardiovascular diseases because the consumption of higher levels of PUFAs is related to reducing cholesterol in the serum and low-density lipids in the plasma [20]. This ratio for the *H. cupressiforme* oil was close to the recommended optimal one, which has been established to be from 0.8 to 1.2 [18]. The index of atherogenicity reveals the relationship between the SFAs (the major ones being lauric, myristic, and palmitic) and the UFAs. The first ones are denoted as proatherogenic, while the second ones are antiatherogenic [19]. The index of thrombogenicity represents the thrombogenic potential of the present fatty acids in the oils and their tendency towards clotting in the blood vessels [20]. The values of the index of atherogenicity and thrombogenicity of the oil from *H. cupressiforme* are below 1.0 which is a sign of the potential of the lipids to reduce the levels of total cholesterol and LDL cholesterol in human blood plasma. The hypocholesterolemic to hypercholesterolemic ratio (HH) shows the relationship between hypocholesterolemic fatty acids (mainly polyunsaturated fatty acids) and hypercholesterolemic ones (saturated fatty acids), and this ratio in the examined oil was higher than 1.0 which indicates the positive effect of the fatty acid composition on cardiovascular disease. In order to establish the rate of oxidation of the examined glyceride oils, the Peroxidability index was also determined, and the Oxidation Stability Index was evaluated through the calculation of the Allylic Position equivalent (APE) and the Bis-Allylic position equivalent (BAPE). The Peroxidability index indicates the relative rate of oxidation and lower values suggest that the respective oil will be less prone to oxidation [43]. The Peroxidability index of the examined moss lipids (57.3) was similar to those of soybean oil (50.33) and grapeseed oil (56.95), and much higher than olive oil (7.10) [43]. The value of APE (84.8) in *H. cupressiforme* oil was almost twice as high as that of BAPE (42.3). It is worth mentioning that a bis-allylic position in the fatty acids is more susceptible to oxidation than an allylic position [23]. The values of APE and BAPE are used for calculating the OSI, the value of which can be used to predict the shelf-life of the lipids [22]. When its value is low, which is the case with the *H. cupressiforme*, it indicates that the oil is a lot more prone to oxidation.

Moss tissue in an in vitro culture is a possible tool for the production of bioactive compounds in large amounts for diverse purposes including pharmaceutical needs. The first study that established stable in vitro culture of *H. cupressiforme* revealed that different developmental stages are stimulated or decreased by various combinations of mineral nutrition, light, and temperature [44].

Another solution is to grow moss artificially in laboratory conditions. Recent research reported moss cultures in plastic containers with organic gardening substrate without fertilizers to achieve an average coverage of the culture area of more than 60% in 5–8 weeks [45].

## 4. Materials and Methods

### 4.1. Materials

**Figure 2:**
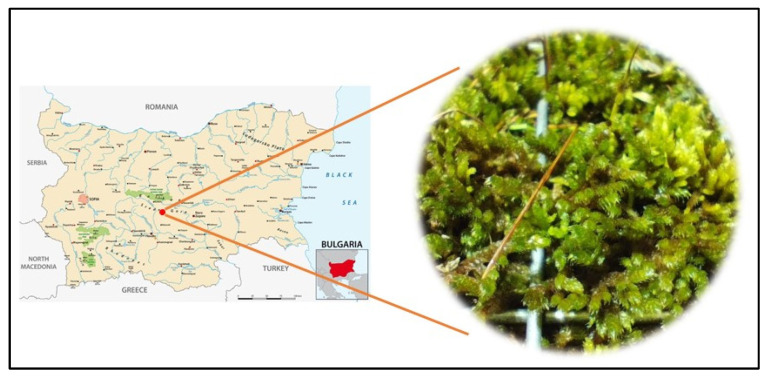
Location of moss *Hypnum cupressiforme*.

*Hypnum cupressiforme* Hedw. was collected from Sredna Gora Mountain, Bulgaria (42°28′06.0″ N 24°56′07.9″ E, altitude of 600 m) (Figure 2). Moss material was sampled from rocks within oak forest clearings in February 2023 in a region representative of a non-urban area. The sampling was done during a period of no rainfall. A total amount for the month of 8 L per square meter was accounted for after the sampling. One composite sample, consisting of ten subsamples, collected within an area of about 50 m × 50 m was immediately subjected to analysis after cleaning the deposited on the moss surface particles.

### 4.2. Chemical Composition

Glyceride oil was isolated with hexane using the extractor Velp scientifica ser. 148. Briefly, three grams of the material were placed in a cellulose thimble and 50 mL of hexane was used for the extraction. The process was set as follows: (1) the duration of immersion was 60 min, (2) the duration of washing was 60 min, (3) the solvent recovery was set to 15 min. The oil content was calculated after evaporation of the solvent.

Total protein content was examined titrimetrically after mineralization of 0.5 g of the sample in the DK6 Digestion unit (Velp scientifica) and following distillation in the UDK 127 Destilation unit (Velp scientifica) [46].

Total carbohydrates were calculated using the following formula [47]:

Total carbohydrates (g/100 g) = 100 − [Oil content (g/100 g) + Proteins (g/100 g) + Ash (g/100 g) + Moisture (g/100 g)](1)

The fibre content was determined after subjecting the material to hydrolysis with a water solution of sulfuric acid followed by potassium hydroxide. Briefly, three grams of the moss were hydrolyzed with 1.25% H_2_SO_4_ for 30 min. Then, the solution was filtered through a filter glass crucible (G2) and washed with hot distilled water. The remaining material was then subjected to hydrolysis with 1.25% KOH for 30 min. After that, the remaining material (as raw cellulose) was filtered again through a filter glass crucible (G2), washed with distilled water, and dried to a constant weight.

Total ash and moisture content were determined according to AOAC [48] and the energy value (kcal/100 g) was calculated as a sum of the proteins, carbohydrates, and lipids (in g/100 g) multiplied by specific conversion factors: 4 for proteins and carbohydrates and 9 for the lipids [47].

### 4.3. Lipid Composition

#### 4.3.1. Sterols

Prior to the analysis of the sterols, the unsaponifiable matter was extracted. Briefly, three to five grams of the oil were subjected to saponification with 2 N KOH in ethanol for one hour. After that, the unsaponifiables were extracted from the solution with 50 mL of hexane and this step was repeated five times [49]. The obtained unsaponifiable matter was diluted in 3 mL of chloroform and 1 mL of the solution was subjected to thin-layer chromatography according to Ivanov et al. [50]. After the isolation of the sterols, their content was determined spectrophotometrically on a Unico UV/Vis spectrophotometer at 597 nm [50].

#### 4.3.2. Tocopherols

High-performance liquid chromatography (HPLC) was used for the total and individual tocopherol determination. Briefly, 40 mg of the oil was dissolved in 2 mL of *n*-hexane. Then, 20 μL of the solution was injected into the HPLC unit (Merck-Hitachi, Burladingen, Germany) with fluorescent detection at 290 nm excitement and 330 nm emission. The column used was Nucleosil Si 50-5 (250 × 4 mm, particle size: 5 μm). The mobile phase was a mixture of hexane and dioxane with a ratio of 96:4 (*v*/*v*) and the flow rate of the phase was 1 mL/min [51].

#### 4.3.3. Phospholipids

The polar lipids were extracted from the moss tissues with a mixture of chloroform and methanol (2:1, *v*/*v*) [52]. After filtration, the residue was dissolved with 50 mL of acetone and put in a refrigerator at −18 °C for one hour and then the solution was filtered through Whatman no. 3 filter paper. The remaining phospholipids were solved in chloroform:methanol (2:1, *v*/*v*) and 0.1 mL of the solution was subjected to a two-dimensional thin-layer chromatography (TLC) to isolate the individual phospholipids [53]. Identification was performed by comparing the Rf values with authentic standards. The spots of the identified phospholipids were scrapped and mineralized with perchloric:sulphuric acid (1:1, *v*/*v*). The quantification was carried out spectrophotometrically at 700 nm [54].

#### 4.3.4. Fatty Acids

The individual composition of the fatty acids present in the oil was determined by gas chromatography (GC) [55]. The oil (100 mg) was subjected to transesterification with 5 mL of methanol in the presence of sulfuric acid to obtain the fatty acid methyl esters (FAME) [56]. They were extracted from the solution with 20 mL of *n*-hexane. Then, the solvent was evaporated on a rotary vacuum evaporator and the dry residue was dissolved with petroleum ether (1% solution of FAME in petroleum ether). After that, 1 μL was injected in the GC unit (Agilent 8860, Santa Clara, CA, USA). The column used was a capillary column DB-FastFAME with the following characteristics: 30 m × 0.25 mm × 0.25 μm (film thickness). The detector was a flame ionization detector (FID) and the carrier gas was nitrogen. The conditions of the analysis were as follows: 70 °C (holding at this temperature for 1 min), then increasing the temperature up to 180 °C at a rate of 6 °C/min, and increasing the temperature up to 250 °C at a rate of 5 °C/min. Identification of the individual FAME was performed by comparing their retention times with that of a standard solution of FAME (Supelco, FAME mix 37 components, Bellefonte, PA, USA). The limit of detection of the GC was 0.5%.

### 4.4. Lipid Indices

#### 4.4.1. Iodine Value

The iodine value (IV, gI_2_/100 g) was calculated based on the fatty acid composition of the oil using the formula [57]:

IV = [(90 × % Oleic acid) + (181 × % Linoleic acid) + (274 × % Linolenic acid)]/100(2)

where 90, 181, 274 are the iodine values of pure oleic, linoleic, and linolenic acids.

#### 4.4.2. Index of Atherogenicity (IA)

The index of atherogenicity (IA) was calculated according to the following formula based on the fatty acid composition [58]:

IA = (C_12:0_ + 4 × C_14:0_ + C_16:0_)/(∑MUFA + ∑PUFA) (3)

In which ∑MUFA is the amount of the monounsaturated fatty acids, ∑PUFA—polyunsaturated fatty acids, C_12:0_—lauric acid, C_14:0_—myristic acid, and C_16:0_—palmitic acid.

#### 4.4.3. Index of Thrombogenicity (IT)

The index of thrombogenicity (IT) was calculated according to the formula given by Ulbricht and Southgate [58]:

IT = (C_14:0_ + C_16:0_ + C_18:0_)/[(0.5 × ∑MUFA) + (0.5 × ∑n-6 PUFA) + (3 × ∑n-3 PUFA) + (∑n-3 PUFA/∑n-6 PUFA)] (4)

In which ∑MUFA is the amount of the monounsaturated fatty acids, ∑n-6 PUFA—polyunsaturated fatty acids (n-6), ∑n-3 PUFA—polyunsaturated fatty acids (n-3), C_14:0_—myristic acid, C_16:0_—palmitic acid, and C_18:0_—stearic acid.

#### 4.4.4. Hypocholesterolemic/Hypercholesterolemic (HH) Ratio

The Hypocholesterolemic/hypercholesterolemic (HH) ratio was calculated by the formula according to Santos-Silva et al. [59]:

HH ratio = [C_18:1_ (n-9) + C_18:2_ (n-6) + C_18:3_ (n-6) + C_18:3_ (n-3) + C_20:2_ (n-6) + C_20:3_ (n-6) + C_20:4_ (n-6)]/(C_12:0_ + C_14:0_ + C_16:0_) (5)

In which C_18:1_ (n-9)—oleic acid, C_18:2_ (n-6)—linoleic acid, C_18:3_ (n-3)—α-linolenic acid, C_18:3_ (n-6)—γ-linolenic acid, C_20:2_ (n-6)—eicosadienoic acid, C_20:3_ (n-6)—dihomo-gamma-linolenic acid, C_20:4_ (n-6)—arachidonic acid, C_12:0_—lauric acid, C_14:0_—myristic acid, and C_16:0_—palmitic acid.

#### 4.4.5. Peroxidability Index (PInd)

The calculation of the peroxidability index (PInd) was carried out using the formula given by Cortinas et al. [60]:

PInd = (% monoenoic FA × 0.025) + (% dienoic FA × 1) + (% trienoic FA × 2) + (% tetraenoic FA × 4) + (% pentaenoic FA × 6) + (% hexaenoic FA × 8)(6)

#### 4.4.6. Allylic Position Equivalent (APE), Bis-Allylic Position Equivalent (BAPE) and Oxidation Stability Index (OSI)

These indicators were calculated by the following formulae described by Kumar and Sharma [22] and Pinto et al. [23]:

APE = 2 × (% C_18:1_ + % C_18:2_ + % C_18:3_) (7)

BAPE = % C_18:2_ + (2 × % C_18:3_) (8)

OSI = 3.91 − (0.045 × BAPE)(9)

where C_18:1_ is the amount (in %) of the oleic acid, C_18:2_—linoleic acid, and C_18:3_—linolenic acid.

### 4.5. Statistics

All of the analyses were conducted in triplicate (n = 3) and the results were given as Means ± Standard deviation (SD).

## 5. Conclusions

The current study presents preliminary results on the chemical and lipid composition of the moss *H. cupressiforme* from a natural habitat in Bulgaria. Based on the obtained results, it was revealed that *H. cupressiforme* is a source of both macronutrients and bioactive compounds that could be implemented in supplements with health-promoting effects and pharmaceutical products. This moss species has a cosmopolitan distribution and can colonise a wide range of habitats. Taking into account the findings in the present research, further studies on the variability of the composition depending on the abiotic characteristics of the habitats will be of great interest. In addition, increasing species biology knowledge and further progress in in vitro culturing will support obtaining the studied valuable compounds.

## Figures and Tables

**Table 1:** Chemical composition of the moss *Hypnum cupressiforme*.

Chemical Composition	Content
Oil content, %	1.27 ± 0.04
Total proteins, %	7.18 ± 0.85
Total carbohydrates, %	76.41 ± 0.93
Fibres, %	30.81 ± 1.53
Ash, %	3.36 ± 0.10
Moisture, %	11.78 ± 0.01
Energy value, kcal/100 g (kJ/100 g)	346 (1469)

**Table 2:** Content of lipid-soluble biologically active components of the moss *Hypnum cupressiforme*.

Biologically Active Components	Content
Sterols, %	
- in the oil	3.2 ± 0.1
- in the tissues	0.041 ± 0.001
Tocopherols, mg/kg	
- in the oil	1848.5 ± 16.3
- in the tissues	23.9 ± 0.2
Phospholipids, %	
- in the oil	50.1 ± 2.8
- in the tissues	0.7 ± 0.1

**Table 3:** Phospholipid composition of the moss *Hypnum cupressiforme*.

Phospholipids	Content, %
Lysophosphatidylcholine	12.5 ± 2.3
Phosphatidylinositol	19.8 ± 2.8
Phosphatidylcholine	19.9 ± 2.5
Phosphatidylethanolamine	15.7 ± 2.1
Phosphatidylserine	17.0 ± 0.6
Phosphatidic acids	15.1 ± 0.7

**Table 4:** Fatty acid composition of lipids isolated from the moss *Hypnum cupressiforme*.

Fatty Acids	Content, %
Saturated fatty acids	43.6
C_4:0_—Butyric acid	1.4 ± 0.1
C_6:0_—Caproic acid	0.7 ± 0.2
C_8:0_—Caprylic acid	8.6 ± 0.4
C_10:0_—Capric acid	0.6 ± 0.1
C_11:0_—Undecanoic acid	0.7 ± 0.1
C_12:0_—Lauric acid	2.1 ± 0.2
C_13:0_—Tridecanoic acid	1.3 ± 0.1
C_14:0_—Myristic acid	4.4 ± 0.2
C_15:0_—Pentadecanoic acid	0.9 ± 0.1
C_16:0_—Palmitic acid	12.5 ± 0.3
C_17:0_—Margaric acid	1.7 ± 0.1
C_18:0_—Stearic acid	6.0 ± 0.2
C_22:0_—Behenic acid	2.7 ± 0.2
Monounsaturated fatty acids	23.9
C_14:1_—Myristoleic acid	0.9 ± 0.0
C_15:1_—Pentadecenoic acid	3.2 ± 0.1
C_16:1_—Palmitoleic acid	1.3 ± 0.1
C_17:1_—Heptadecenoic acid	2.8 ± 0.2
C_18:1_—Oleic acid	13.8 ± 0.4
C_20:1_—Eicosenoic acid	1.9 ± 0.1
Polyunsaturated fatty acids	32.5
C_18:2_ (n-6)—Linoleic acid	14.9 ± 0.5
C_18:3_ (n-6)—γ-Linolenic acid	2.4 ± 0.2
C_18:3_ (n-3)—α-Linolenic acid	11.3 ± 0.2
C_20:3_ (n-6)—Dihomo-γ-linolenic acid	0.6 ± 0.1
C_20:4_ (n-6)—Arachidonic acid	3.3 ± 0.2

**Table 5:** Lipid indices of glyceride oil from the moss *Hypnum cupressiforme*.

Lipid Indices	Values	Legend (Optimal Values)
Iodine value, gI_2_/100 g	76.9 ± 2.8	<100—Non-drying oil [17]
PUFA/SFA	0.7454 ± 0.0443	Optimal ratio: 0.8–1.2 [18]
Index of atherogenicity	0.5709 ± 0.0006	<1.0—indicates better anti-atherogenic properties of the oils [19]
Index of thrombogenicity	0.4019 ± 0.0098	<1.0—indicates better anti-thrombogenic properties of the oils [19]
Hypocholesterolemic/hyper-cholesterolemic (HH) ratio	2.4368 ± 0.0128	>1.0—indicates better hypocholesterolemic potential of the lipids [20]
Peroxidability index (PI)	57.3 ± 1.5	Low values: higher stability of the oils (indicates the relative rate of the oxidation) [21]
Allylic Position equivalent (APE)	84.8 ± 3.0	indicates the presence of -H_2_C=CH-CH_2_- [22]
Bis-Allylic position equivalent (BAPE)	42.3 ± 1.6	indicates the presence of methylene-interrupted double bonds in the hydrocarbon chain of the fatty acids (R-CH=CH-CH_2_-CH=CH-R) [22]
Oxidation Stability Index (OSI)	2.0 ± 0.1	indicates the rate of oxidation of the lipids [23]

## Data Availability

The data presented in this study are available on request from the corresponding author.
